# The Oglala Sioux Tribe CHOICES Program: Modifying an Existing Alcohol-Exposed Pregnancy Intervention for Use in an American Indian Community

**DOI:** 10.3390/ijerph13010001

**Published:** 2015-12-22

**Authors:** Jessica D. Hanson, Susan Pourier

**Affiliations:** 1 Sanford Research, 2301 E. 60th St North, Sioux Falls, SD 57104, USA; 2 OST CHOICES Program, PO Box 824, Pine Ridge, SD 57770, USA; choicescoordinator@gmail.com

**Keywords:** alcohol-exposed pregnancy, American Indians, alcohol, birth control, program development

## Abstract

Alcohol-exposed pregnancies are a health issue for many American Indian communities. The goal of this manuscript is to outline how an existing alcohol-exposed pregnancy prevention program with non-pregnant women (Project CHOICES) was modified to fit the needs and norms of an American Indian community. The Oglala Sioux Tribe CHOICES Program was developed and implemented using community feedback through initial meetings, reviewing materials, gathering input into recruitment and intervention logistics, and conducting interviews to evaluate the program. The intervention was implemented and has been enrolling non-pregnant American Indian women for the past several years. While data collection is ongoing, it has shown preliminary success in changing behaviors and in impacting how the community views the prevention of alcohol-exposed pregnancies. Overall, this study highlights the potential to expand this prevention program to other sites and with other populations, such as adolescents. By the end of this article, readers will comprehend the steps necessary to replicate such a program at other tribal and rural sites.

## 1. Introduction

Fetal Alcohol Spectrum Disorders (FASD) is the continuum of lifelong outcomes in those born prenatally exposed to alcohol and includes a diagnosis of fetal alcohol syndrome (FAS) [1]. FAS, the most physically recognizable outcome, is characterized as having facial abnormalities (*i.e.*, palpebral fissures, thin vermilion, smooth philtrum); evidence of growth retardation; and evidence of delayed brain growth [2,3,4]. In addition to physical features, prenatal exposure to alcohol is linked to conduct disorders (*i.e.*, delinquency and aggressiveness), mental illness (*i.e.*, depression, anxiety disorders), and psychosocial functioning [5,6,7]. 

FASD is especially concerning for American Indian communities, although it is by no means unique among this population [8]. In a previous study, Indian Health Service (IHS) reported that up to 56% of pregnant American Indian patients reported drinking alcohol during pregnancy [9,10], while a recent national study found that 7.6% of pregnant women drank any amount of alcohol and 1.4% binge drank, compared to a national study from ten years ago where 2% and 5% of women reported binge drinking during pregnancy and 10-13% of pregnant women consumed moderate amounts of alcohol [11,12]. Rates of FAS among Northern Plains American Indians range as high as 9 per 1,000 births [13], although there have been few recent studies on the surveillance of FAS or FASD in American Indian communities. Among the general population, a recent study utilizing active case ascertainment to examine FASD among first grade children in an Upper Midwest city found the rate of FAS in this community to be 5.9 to 10.2 per 1000 children [8].

Traditionally, interventions to prevent FASD have focused on pregnant women, although recent research concludes that prevention of FASD must begin preconceptionally, or before a woman even becomes pregnant, by either reducing alcohol consumption in women at-risk or planning pregnancy, or preventing pregnancy in women drinking at risky levels [14]. Studies have shown between 10% and 26% of sexually active women are at-risk for AEP [15], and in many American Indian tribal communities, rates are higher. For example, a previous project with three Northern Plains tribes found among a population of women drinking at risky levels, nearly 30% were not using birth control to protect against pregnancy [16], meaning they were at risk for AEP. Another study from the South Dakota Tribal Pregnancy Risk Assessment Monitoring System found that 43% of American Indian women surveyed were binge drinking in the three months prior to pregnancy [17]. Among this same sample, 65% who were sexually active (but *not* trying to get pregnant) were not using any birth control at conception. 

One AEP prevention program currently underway with non-pregnant American Indian women is the Oglala Sioux Tribe (OST) Changing High-risk alcohOl use and Increasing Contraception Effectiveness Study (CHOICES) program. This is based on the original Project CHOICES curriculum focused on reducing risk for AEP through alcohol reduction and pregnancy prevention using an in-person brief intervention and motivational interviewing sessions with non-pregnant women at-risk for AEP [15,18,19,20,21,22]. Motivational interviewing is a counseling style that “guides the individual to explore and resolve ambivalence about changing [behavior], highlighting and increasing perceived discrepancy between current behaviors and overall goals and values” [19]. The original Project CHOICES participants received four face-to-face motivational intervention sessions, plus a separate contraception counseling session. The focus of the intervention was on self-guided change, where the participants themselves set behavior goals and worked with the interventionists to assess readiness to change. The original CHOICES intervention significantly decreased the risk of an AEP in the intervention group [18]. 

The CHOICES theoretical model was previously implemented with OST and two other tribes through a five-year project that utilized a telephone-based enrollment and participation [23], personalized feedback, and a workbook based on self-guided change constructs. Follow-up phone calls were conducted every three months for one year. A total of 230 AI women were included in the analysis. Baseline drinking among the participants averaged 7.0 drinks per occasion, and 30% of those who were sexually active used no contraception during sex. Data analyses indicated that participants had significant decreases in alcohol consumption, and there was a significant increase in those using protection from baseline to the three month follow-up. However, there was a fairly high loss to follow-up rate [16], and it was felt that a face-to-face intervention over a shorter time period might yield more participation [19]. 

Therefore, OST took data from this study to develop its current OST CHOICES Program, beginning by modifying CHOICES materials and piloting the CHOICES intervention at tribal clinics. The goal of this manuscript is to outline how an existing AEP prevention program (CHOICES) was modified to fit the needs and norms of an American Indian community. We also present preliminary data on the success of the OST CHOICES Program in lowering risk for AEP with non-pregnant American Indian women. 

## 2. Methods

The OST CHOICES Program began in September, 2010 by working with three clinics, two located on the reservation and a third that serves American Indian women in an urban setting. There were two major stages to this program that will be reported here: first, an evaluation of the feasibility and acceptability of the CHOICES intervention with a tribal community, and second, the actual implementation of the program, highlighted through preliminary data analysis. 

To begin the first stage, initial meetings were held with clinic staff at the sites to introduce the project and to discuss questions and concerns. The initial questions involved clearly defining the benefits of this project for the patients and the tribe. The clinics felt the intervention should be conducted by someone already working within the clinics or a tribal member who the women would be comfortable interacting. These types of meetings were held regularly to get input on the development and implementation of the CHOICES curriculum.

In addition, project staff met with the clinic directors and staff to review the CHOICES materials in an informal group setting where OST CHOICES staff went through all the materials with the CEOs of the clinics involved, nursing/midwife staff, clinical directors, and an administrative officer at one of the clinics. The focus of these meetings was to suggest alterations to make the CHOICES materials more locally appropriate. For example, the clinics wanted local images added to the intervention materials, an acceptable readability, and to make information local (*i.e.*, local statistics on how many pregnancies are unplanned). Similarly, information in the materials had to be changed to fit with the most common types of alcohol consumed in the communities, and certain types of birth control information had to be taken out, as the clinics did not offer certain methods of contraception.

Next, the implementation of CHOICES into tribal clinics involved gathering input into recruitment and intervention logistics. Clinic staff was actively involved in figuring out recruitment strategies. For instance, one of the sites utilized a CHOICES brochure and “word of mouth” to encourage recruitment. At another site, recruitment occurred via flyers and newspaper ads, as well as receiving referrals from the clinic’s midwife. At one of the sites, there was an interest in CHOICES but staff was unable to implement the curriculum because of a shortage in providers at the time. However, this site was still involved in CHOICES by referring women to the other CHOICES sites. It was also the clinics that identified who would be best to conduct the intervention. For example, a nurse practitioner showed interest in being the interventionist at one site, while a behavioral health specialist was involved at another site. 

Finally, in order to better evaluate the efficacy and sustainability of the CHOICES program at the sites, the evaluation team conducted qualitative interviews with the CHOICES interventionists at the two initial CHOICES sites, as well as with a clinic manager at one of the sites. Open-ended questions were utilized to ask these key staff about their thoughts on preventing AEP with non-pregnant women; suggestions for improving the CHOICES; barriers and successes in implementing the intervention; success stories with the CHOICES participants; and how they envision sustaining the CHOICES program in the long-term. 

We then moved to the second stage of implementation. Based on this extensive process of gathering community and clinic input, the OST CHOICES intervention was implemented and has been enrolling participants since 2012. At one site, American Indian women in the OST CHOICES program see the interventionist for four motivational interviewing sessions, while at two others they receive two sessions, per the preference of the site and stakeholder input. At the CHOICES sessions, participants set goals for their drinking and contraception behavior. They also complete daily diaries that track their drinking, sexual activity, and contraception use, and work with the interventionist to define their readiness to change these behaviors. After the sessions and the separate birth control session, the CHOICES interventionist follows up with women at three and six months to evaluate if they sustain behavior changes related to AEP.

## 3. Results

### 3.1. OST CHOICES Feasibility and Acceptability

The first two years of the project (2010–2012) were dedicated to adapting the CHOICES materials for AI women and evaluating the feasibility of the CHOICES components with this population. Overall, the staff at the three tribal clinics involved in this first stage were extremely enthusiastic about the program and the potential for reducing AEP, and there was a willingness to implement the CHOICES curriculum within the clinic protocol. Input was gathered on modifying the CHOICES curriculum to make it appropriate for the tribal partners, including adding culturally appropriate images, checking the materials for readability, ensuring that data (such as rates of unplanned pregnancy) were local rather than national data, and determining the relevance of the information, such as what birth control options are available locally and the types of drinks that women drink. 

While the OST CHOICES Program began in September, 2012, it began slowly because of various implementation issues. One of the sites dropped out of participation and declined to enroll women because of a staff shortage, meaning that although they were interested in the OST CHOICES Program, they were unable to dedicate any staff time to enrolling participants into the program. Another site began enrolling women but the interventionist soon left the program and no follow-ups were conducted. Staff turnover was a concern, although that challenge is now overcome because both authors of this manuscript, who are key staff with the OST CHOICES Program, attended a national CHOICES “training of trainers” and are now able to train any new staff.

However, the results of the qualitative interviews with two of the OST CHOICES interventionists and other relevant clinic staff point to the positive outcomes of the intervention in this feasibility/acceptability stage. These interviews revealed that the focus on preventing AEP with women that aren’t currently pregnant is an ideal prevention route. Most of the women enrolled appear to like the birth control part of this effort. The interventionists also feel that the approach is non-judgmental, which resonates with the women they see. The largest success stories include young women with multiple children who begin using birth control, in particular long-term birth control methods. When asked about sustaining the CHOICES intervention, one suggestion was to make CHOICES an official Indian Health Service program so that all American Indian women would be screened for alcohol consumption and pregnancy risk and would automatically be referred to the local CHOICES interventionist to enroll. 

### 3.2. OST CHOICES Implementation

**Figure 1:**
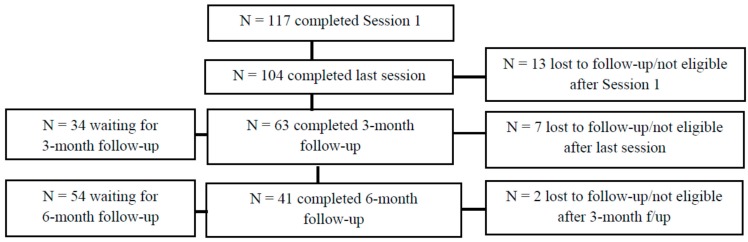
OST CHOICES program: individual session data.

Based on the positive input from the OST CHOICES interventionists, two additional sites were added in 2013 with the advent of further funding for CHOICES for a total of three clinic sites, and their data is included here. While enrollment and follow-up is ongoing, as of June 18 2015, 117 women have been enrolled at these three sites for the individual CHOICES sessions (see Figure 1). All participants were at-risk for an AEP because they were binge drinking and were sexually active and not using effective birth control. Note that “waiting for [3- or 6-month] follow-up” in Figure 1 indicates that the participant has not completed the follow-up because they have not reached that time point yet. 

As stated earlier, during these CHOICES sessions, MI techniques are used along with various activities, including decisional balance exercises, goal setting, and behavioral change plans. One component of the OST CHOICES intervention includes asking the participant to identify a person that can help in reaching behavioral goals and ways they can help. See Table 1 for examples. In addition, participants were asked to complete “decisional balance” exercises to reflect about the good and not so good things about their drinking alcohol and use of birth control. Table 2 includes just a few responses from the participants enrolled in OST CHOICES. 

Finally, participants were asked to set goals for their drinking and birth control use and reflect on why they chose their particular goal. Women said powerful things about their alcohol goals, such as, “*To start building confidence in myself”; “I’m still young and I just drink all the time”* and “*Because I want to be here for my children living an alcohol/dry free life.”* To change their drinking, they noted steps they needed to take, including keeping the empty bottles or tabs by them to see how much they’ve been drinking, being around friends and family who don’t drink and avoiding friends that do, and keeping busy with work and school. Participants also discussed cultural elements such as praying, going to sweats, smudging, and engaging in ceremonies to get healthy. Regarding using birth control, they said powerful things about using birth control, such as, “*Because I believe I am a risky drinker and I don’t want any more children”; “My life as a young adult can be at stake. My life is at stake. I’m not ready”* and *“Because I do not want STDs or a baby with FAS”.* At the final assessment, when asked to make a final goal statement about alcohol use, participants made goals to not binge drink, to socialize with friends and family that do not drink, attend alcohol treatment, AA, or other support meetings, and to look at their children as motivators in avoiding drinking. One participant said, “*I plan to not give up on myself.*” See Table 3 for some final thoughts from OST CHOICES participants on the intervention.

**Table 1:** Identified social support person and impact on behaviors.

Individual	How/Why They Impact Drinking	How/Why They Impact Birth Control
Mother	Keeps woman focused on other things and supports. She doesn’t drink/doesn’t like drinking and gets mad.	Reminds her to avoid unplanned pregnancy Will drive her to appointments.
Older female relatives/female elder	Dislikes drinking and worries about woman. Talks about consequences and keeps her focused.	Reminds her about birth control. Supports woman no matter what.
Young female relative (sister, cousin)	Don’t drink as much or at all (against drinking). Provide encouragement and confidence to be sober.	Reminds of difficulty of having children. Go with/remind about birth control appointment.
Boyfriend/male partners	He doesn’t drink/don’t buy alcohol for participant. Positive support and supports alternative activities.	Go with to appointment for support. Talk about birth control options.

While enrollment and follow-up is ongoing, as of June 18, 2015, 117 women have been enrolled at these three sites for the individual CHOICES sessions (see Figure 1). Because women were self-selected, none refused to participate. All participants screened positive and were at-risk for an AEP because they were drinking at risky levels, were sexually active, and were not using effective birth control. We lost a total of 22 participants (18.8%) from baseline to the last follow-up session (of note, there are no significant differences in those that were lost to follow-up *versus* those that were not), and some participants have not yet received their follow-up sessions. Of the 63 women reached for the *3-month follow-up*, 42 (66.7%) were at reduced risk for an AEP because of their use of birth control (60.9% of the total who completed follow-up), reduced alcohol consumption (4.4%), or changes in both behaviors (34.7%). At the *6-month follow-up*, of the 41 reached, 31 (75.6%) were at reduced risk for AEP either because they began utilizing birth control (67.7%) or both began using birth control and reduced alcohol consumption (32.3%). The team is encouraged by the results, as preliminary data analyses indicate that the intervention successfully reduced risk for AEP in the majority of participants.

**Table 2:** Decisional balance exercise responses.

Behavior	Good Things	Not So Good Things
Alcohol	Forget problems; don’t feel the pain, in a better mood. Have a good time. To celebrate. Be with friends and make new friends. Happier, more outspoken and outgoing.	Cost/no money. Family is sober and dislikes her drinking. Going to jail, other problems with the law, like DUIs. Problems are still there. Can ruin friendships. Hangovers/feeling sick and tired the next day. Fighting or getting hurt while drinking. Dirty house. Not taking care of kids/being away from children for days. Smoking cigarettes/weed when drinking. Missing work. Blacking out/not remembering things. Unprotected sex/embarrassed about a sexual encounter. Being sad/remembering people who passed away. Losing cell phone and other things while drinking. Causes health issues. Sad and lonely when she sobers up.
Birth control	Fixes abnormal menstrual periods. Prevents STDs. Birth control free at IHS. Pregnancy prevention.	Weight gain. Irregular periods or bleeding between periods. Have to use method all the time (pill, condoms). Men don’t always have condoms with them. Condom could break/safety of condoms. Remembering to take the pill. Sometimes birth control isn’t available or can’t afford birth control. Fighting with boyfriend about birth control.

**Table 3:** Opinions of OST CHOICES participants on the intervention.

“To control my habit and to be realistic about goal setting, to be more careful about my sexual activities to practice safe sex. Alcohol is a bad habit for me and I need to slow down because I am always the one hurting myself and it enables me to be active with my daughter. So cut back and believe in myself that I can do this and accomplish my goals, maybe in time I will be alcohol-free.”
“That my alcohol use affects a lot of different aspects of my life. It affects my health, my financial stability, my family. I’ve learned that if I cut back on my drinking, I could do more positive things with my children, I could save a lot more money if I didn’t drink (so much). I don’t have to worry about things I may have done while drunk and impaired, I've learned that I can control my own actions and alcoholism if I really wanted to.”
“I’ve learned so much from CHOICES, the awareness of alcohol and unsafe sex and just getting the education of both is reality. It has taught me to talk to my nieces, cousins, daughter that it’s really important. CHOICES is the best education and prevention of also STD's, drinking too much. I've learned a lot.”
“I am thankful for CHOICES because they taught me a lot about drinking and the effects it had on my child. Thanks to the program I am now going to AA classes and I now have a better view on the effect of alcohol on a baby.”

## 4. Discussion

The OST CHOICES Program was modified from the original CHOICES program through extensive community and clinic input, highlighting that CHOICES is feasible within a tribal clinic setting, keeping in mind that formative work is necessary to make the program’s materials culturally and geographically appropriate. The intervention has been enrolling non-pregnant American Indian women for the past several years and has shown preliminary success in changing behaviors and in impacting how women as well as practitioners view prevention of AEP. Our study also showed that the CHOICES intervention was an acceptable and even welcomed intervention by this particular tribal community in order to prevent AEP, and its success shows that it can be implemented with other interested populations. In fact, there is interest from other tribes in seeing the CHOICES curriculum developed and implemented in their communities. Two staff members from the CHOICES team were trained to provide the CHOICES curriculum training (*i.e.*, training of trainers), meaning that the expansion of CHOICES to other tribal sites and clinics should be fairly economical and straightforward. In addition, while the process to modify and implement the CHOICES curriculum within this community was fairly methodical and relatively time consuming, the implementation in other communities need not be. The CHOICES curriculum is free and available online, therefore communities need only make community-based modifications as they see fit, if at all. 

Overall, the OST CHOICES Program is unique because it is a *tribally-run program* that has worked in collaboration with a research center for the past several years. Using a community-based participatory research (CBPR) approach, we have balanced research and programmatic action for the mutual benefit of our partners and the women we work with. Similar to a previous study by Masis and May (1991), American Indian women at-risk for an AEP can lower their risk by either reducing alcohol consumption or preventing pregnancy, or as is often the case, by doing both [24]. The OST CHOICES Program is also unique from previous studies focused on preventing FASD because it targets non-pregnant but at-risk women, highlighting the importance of primary prevention efforts, especially by increasing utilization of birth control, in the reduction of AEP risk. This type of work and intervention is also significant because it focuses on a demographic (American Indians) that are often left out of programmatic research because of its relatively small population.

What makes the evidence-based CHOICES intervention distinctive in the prevention of AEP is that it targets more than one risky behavior related to AEP, reducing risk for AEP either by increasing a woman’s use of contraception or by decreasing alcohol consumption. The women in our study typically focused their behavioral efforts on obtaining and utilizing an effective form of birth control rather than decreasing alcohol consumption or addressing both contraception and drinking. Although our OST CHOICES data is preliminary, our initial analysis indicates that the AI women in our program are more willing to begin utilizing contraception to prevent pregnancy rather than reducing alcohol consumption. This compares to the original CHOICES study, which included a randomized controlled trial, where participants were more equally distributed in their ways of reducing risk for AEP [18]. Specifically, at the three month follow-up, 33.8% of CHOICES participants reduced AEP risk by utilizing contraception, 27.6% reduced drinking, and 38.6% of women used both effective birth control and reduced drinking. At the 9-month follow-up, over 47% of participants were both using birth control and had reduced drinking. 

While the OST CHOICES Program was successfully implemented and enrollment and data collection is ongoing, our team saw a critical need to expand the program to include components the community deems important. First, we conducted a validity/reliability study to further our efforts on the expansion of the CHOICES intervention with American Indian women [25]. Second, we conducted a needs assessment in 2013 that identified several ways to expand OST CHOICES, including a desire to include interpersonal social support in the intervention [26]. To meet this community need, we are in the process of piloting the CHOICES intervention in a group setting, a methodology that utilizes cultural norms of group communication and provides support through group interaction [27]. Through our community engagement, we also identified a need to work with adolescents and young American Indian women to expand the population we work with, and we have applied for funding to expand to that group [28]. With this important community input and with extensive opportunities for expansion, the OST CHOICES Program will only grow in the coming years and expand to serve additional women in the movement to prevent AEP.

### Limitations

There are a few limitations to this study. First, the development, implementation, and evaluation of preliminary results are confined to one American Indian tribe and one urban community; therefore, the results cannot be generalized to all American Indians/Alaska Natives. In addition, we have a relatively small sample size, although data is ongoing. Also, as with many intervention projects that include follow-up, we did have participant turnover for a variety of reasons (*i.e.*, women no longer interested in participating or their contact information has changed), and our staff continues to work diligently to contact participants via both telephone and letter. Finally, our project has faced staff turnover and a clinic dropping-out of data collection, which although created difficulty in the short-term, added to understanding the feasibility of CHOICES within a tribal community. We were able to add a new site and train additional interventionists to overcome these set-backs. As stated earlier, two of our staff members attended a CHOICES “training of trainers” so that we are able to train new staff in-house, meaning that staff turnover can be quickly addressed. Our staff learned a great deal in developing and implementing the OST CHOICES Program and those lessons learned should assist in sustaining the program long-term. 

## 5. Conclusions

Overall, the methods and results of this study highlight the potential to expand an AEP prevention program to other sites. Garnering community input and appropriately modifying materials has led to a successful AEP intervention within an American Indian community. Ideally, those involved in future CHOICES implementation efforts can utilize the methods and results discussed above to implement and sustain this important AEP prevention program.
